# The Bandage in the Treatment of Fractures, Ulcers, Gun-Shot Wounds, Hemorrhage, &c., &c.

**Published:** 1850-02

**Authors:** Theodore S. Bell

**Affiliations:** Louisville, Ky.


### WESTERN JOURNAL

OF

MEDICINE AND SURGERY.

---

FEBRUARY, 1850.

---

### ART. I.—*The Bandage in the Treatment of Fractures, Ulcers, Gun-Shot Wounds, Hemorrhage, &c., &c.* By THEODORE S. BELL, M. D., of Louisville, Ky.

In a former number of this Journal we examined, very briefly, a paper by Dr. Stone, surgeon to Bellevue Hospital, New York, on the subject of amputations, gun-shot wounds, &c. The laudable spirit manifested by Dr. Stone in his careful endeavor to ascertain the best and most successful methods of treatment, for certain forms of injury that require the aid of surgical science and art, commanded our admiration and respect. Experience and observation have taught us a better resource of surgical art than that which he commends, but his efforts at improvement upon the common methods are worthy of praise.

In order to forward the attempt to find the best resources of surgical art, we purpose to examine the use of the roller, in its application to various diseases and injuries. Why it should be neglected to the extent that it is, we cannot conceive. Some of the highest authorities in surgery have spoken in the strongest terms of it, have fully explained the principles of its action, and borne testimony to its great merits. These same authorities have carefully laid down rules for procuring the requisite pressure, and taught us how to apply the roller. And the experience and observation of every man who understands the science of the roller, and applies it properly, must give assurance that while, in all cases where it may be used, it can accomplish all the useful results of other agents, it can claim, as its own right, benefits not to be acquired through any other agency. But it is strange that an agent recommended by such a surgical authority as John Bell, and enforced, as it was by him, with a clear appreciation and explanation of its multifarious powers, and which has surely never failed yet, in the hands of a surgeon capable of using it properly; it is strange, we repeat, that it should have commanded so little respect in great works on surgery. Dr. Cutler, a staff-surgeon in the Belgian army, undertook some time ago to prepare what he called "The Surgeon's Practical Guide in Dressing, and in the Methodic Application of Bandages." He said, in the prefatory remarks to his work, that "a manual, calculated to improve a department of surgery which unfortunately has been too long neglected in this country, and spare the practitioner the sacrifice of much valuable time, cannot but be deemed a great desideratum." We hoped for much from this introduction, but found that "the promise had been held to the ear, and broken to the hope." The object of the author seems to have been to crowd his work with a mass of contrivances, based on no sound principle, and to adorn his pages with a variety of wood cuts. Less than half the space occupied by his details would have sufficed to give a clear and scientific account of the bandage, which might have justly entitled the work to be called the "Surgeon's Practical Guide." We have the simple roller cut up, on Dr. Cutler's pages, into the circular, spiral, reversed, uniting, dividing, compressing, expelling and retaining bandage, with but little practical matter on the subject. These various forms are adapted, according to Dr. Cutler, "to incised wounds, burns and wounds attended with great loss of substance, for preventing the formation of seams or unsightly cicatrices; sprains, edematous swellings, callous ulcers, varices, aneurisms, erectile tumors, ordinary ulcers, deep-seated abscesses, contused wounds, prevention of the formation of sinuses, fractures, dislocations, &c." It is strange that Dr. Cutler did not give the philosophy of the action of an agent in surgery, capable of the extensive application and utility he ascribes to the bandage. John Bell, and Dr. Baynton and Mr. Whately had pointed out the path of this philosophy so clearly that it should have been easily followed, in preparing "The Surgical Guide," in "the methodic application of bandages." Dr. Cutler seems to have caught a glimpse of the light blazing around him; he says: "a bandage is useless which does not give most perfect support to the parts, maintain them in the position necessary to ensure the fulfilment of the indication proposed, and exert on the member an equable compression: for this, they require sometimes to be moistened, which will occasion them to sit more firmly. When applied too tight, or when the compression is not uniform, very serious consequences arise, such as edematous swellings, and even mortification, &c." Of the value of quietude in a reparative process, Dr. Cutler seems to have clear ideas, yet he cannot divest himself of those follies that are in vogue, such as the application of medicated liquids, oils, ointments, escharotics, &c., in connection with the bandage treatment. We hope to be able to show that the surgeon who is familiar with the principles of the bandage treatment, and who understands its application, may rid himself and his patients of a mass of the useless "sovereign'st things" that cumber the pages of surgical works.

In order to show that we do not place an over-estimate upon the bandage, we quote Dr. Cutler's testimony to its value, even with his limited and often erroneous views of its use. He says:

"In the department of surgery, which constitutes the subject of the present work, more perhaps than any other, is the practitioner's reputation exposed to the severity of criticism; and on the degree of knowledge and dexterity which he evinces in this, to be attested by the greater or less acuteness of the patient's sufferings, the duration of the treatment, and the issue of the case, will be found to depend the favorable or unfavorable general opinion of his talents: the majority of people can only appreciate what is palpable to the senses in the practice of the healing art, and therefore it is not surprising that they exercise their privilege of criticism to its utmost limits here."

That these considerations should have some weight with the surgeon, provided the patient does not suffer, while the surgeon is furthering his own glorification, may be admitted; but they should be of minor concern when compared with that important matter—the selection of the best means for the case in hand. We are confident that the bandage, for support and compression, is infinitely before all other methods of treatment, in a great range of surgery, and if it is so, that constitutes the valid reason for its use. It requires study, practice and judgment, but no one who resorts to those means of perfecting himself, will ever feel that he has wasted means that belong to a noble art.

The experience we have enjoyed in the practice of the bandage, often makes us feel a sense of humiliation in reading the works of the great masters of surgical science and art. Take, for example, one of the master minds of modern surgery—the Baron Larrey, a gentleman who enjoyed opportunities for improving his art, such as few ever possessed. He was the pupil of Sabattier; he accompanied Napoleon in the Egyptian campaign, and was a favorite of that illustrious man in all his succeeding campaigns. For his excellent services, he was made a Baron, after the battle of Wagram, and enjoyed the unlimited confidence not only of Napoleon, but of the soldiery. Of his eminent abilities as a surgeon, and of his virtues as a man, no one can entertain a more exalted opinion than we do; yet, it seems to us that a more empirical practice than that commanded by Larrey, at Dresden, for the ulcerations arising from congelation, among the victims to Napoleon's disastrous retreat from Moscow, could not have been adopted. The obvious demands of these cases were something of a supporting character, applied to the part suffering from congelation, not only to re-invigorate it, but to stop the ravages of decay. There is nothing in the hands of surgery that can compare to the bandage for the fulfilment of these palpable indications; in a long experience of its powers in this respect, we know of nothing that can offer such gratifying results, in the treatment of ulcerations of this character, as the simple roller. Yet, in the circular addressed from Koningsberg, by Baron Larrey, surgeon-in-chief to the Imperial army, to Mr. Bancel, we find the following language: "Indeed, parts disorganized by caustics, or by cold, form a gangrenous eschar of greater or less thickness, the removal of which it is necessary to promote by topical applications, *supporting the action of parts that remain sound, and at the same time softening the parts laboring under gangrene.*"

A long time prior to this, Underwood had insisted upon the important uses of the bandage in the treatment of ulcers, and Whately and Baynton, in 1799, had triumphantly established the immense superiority of the roller over every other method of care that had ever been attempted. To these gentlemen the medical profession is deeply indebted for the great improvements introduced by them, into the treatment of ulcers. If Baron Larrey had impressed upon his staff the principles laid down by Dr. Baynton, he would have found in a calico roller, and strips of adhesive plaster, more virtue in the treatment of the ulcers resulting from congelation, than in all the storax and saffron ointment, and charpie, that could have been manufactured in France. If the unrelenting Berlin and Milan decrees, in their war upon English manufactures, deprived the "grand army" of the benefits of the bandage in the treatment of its wounds, we may look upon their action as an illustration of the rattlesnake inflicting its own venom upon itself. The adhesive strip and roller would have saved many a limb that was destroyed by gangrene, and many a life that was undermined by the same disaster. There is nothing, whatever, that so effectually strengthens the debilitated sub-systems, concerned in gangrene, and so perfectly secures reparative secretions, instead of gangrenous fluids, as the bandage. We have seen enough of the display of its powers to make us think that its failure is almost impossible, and we have often seen it succeed after all other means had failed.

In looking at the cartloads of lotions, unguents, poultices and *appareil* commended by the masters of surgical art, for the management of those cases, for which the plainest dictates of common sense would resort to the bandage, and then turning to the impressive eloquence of John Bell on the benefits of the roller, we are surprised that that agent ever lost its merited position in surgery. What a commentary upon apothecarean and culinary surgery does one statement of John Bell make! He says: "In a diseased bursa, as in a relaxation of the knee-joint, that disease which with but a little indulgence, a very little encouragement of fomentations, poultices, bleeding, low diet, would end in white-swelling of the knee, may be stopped even by so simple a matter as a well rolled bandage." This truth, of the supporting and regulating influence of the bandage, may be made to assume a comprehensive position towards an extensive class of surgical cases, wherein the miserable contrivances, mentioned by John Bell, are used with the disastrous results referred to in diseases of the knee-joint.

We know of no author who has prescribed the bandage with more scientific knowledge than John Bell. He often loses sight of his own truths, but this does not destroy his truths. His enlarged understanding surveyed the whole field of vision, and pointed out the most successful methods. The opinion, for instance, is entertained among a few surgeons, who have lost sight of John Hunter's accurate observations, that a gun-shot wound must slough throughout the track of the ball, but those who use the bandage, properly, know that this is not necessarily true. On this point, John Bell says: "It is particularly to be observed, that, in gun-shot wounds, and other bruised wounds, though it would be imprudent to sew the parts, since it is impossible they should altogether unite, *yet the gentle and general support which we give by a compress and bandage, prevents them from separating far from each other, unites the deep parts early, and lessens the extent of that surface which must necessarily fall into suppuration.*" In this extract, the great principle of the bandage is developed, in that kind of surgery to which it is generally considered most inapplicable. But under the influence of the bandage, we have seen gun-shot wounds heal by the first intention. We once succeeded in producing this result in a case where the ball entered the skin over the symphysis pubis, passed around one-half of the pelvic wall, and came out through the skin covering the lumbar vertebra.

The power of the bandage in the suppression of hemorrhage is powerfully enforced by John Bell, and there is no department of surgery in which its virtues are more conspicuous. In an experience of nineteen years, in a great many cases of formidable hemorrhage, we have relied upon compresses and the bandage alone, and never have tied an artery for hemorrhage, except after amputations, and in one case where the brachial artery was severed near the axilla, in a remarkably weak and delicate child, who could not have borne the bandage. John Bell's remarks, contained in a single paragraph, cover the entire ground of the uses of the bandage in hemorrhage, and of the principles of its application in all cases. He says: "If a compress be neatly put upon the bleeding arteries, if there be a bone to resist the compress, or even if the soft parts be firm below, and the bandage be well rolled, the patient is almost secure. *But such a roller must be applied smoothly from the very extremities of the fingers or toes; the member must be thoroughly supported in all its lower parts, that it may bear the pressure above. It is partial stricture alone that does the harm, creates intolerable pain and anxiety, or brings on gangrene.*"—Hemorrhagy requires a very powerful compression, which must, therefore, be very general, and must be very cunningly and skilfully made, to be either supportable or safe; it must not be made only over the bleeding arteries, which is all that the surgeon thinks of in general, nor must it be begun at that part where it is particularly required; the bandaging, for example, by which a wounded artery at the bending of the fore-arm may be cured, must be begun at the very tips of the fingers; each individual finger must be rolled; the roller must be continued over the hand, with the greatest attention to leave not a single point unsupported, nor subject to strangulation. It must be rolled carefully and firmly upwards along the fore-arm and thus the whole of the limb will be supported against that pressure which is made, particularly upon the wounded part. *When thus rightly applied, the firmer the bandage is, the less apt it is to be attended with danger.* Gangrene is, you may easily perceive, the effect, not of a firm bandage because it is firm, but because it is partial, and strangles some single point of the limb."*

This eminent surgeon also bears testimony to the fact, that "at one period of surgery, the bandage took the place of every other method of suppressing hemorrhage." It is difficult to assign any reason for the abandonment of an agent, not only of uniform success, but so infinitely preferable to the ligature, in every point of view.

Nor is it easy to account for the silence of a multitude of surgical works, professing a devotion to the opinions of John Hunter, on the character of an agent capable of exercising the extensive influence that the bandage does, over a vast range of surgical affections, through its control of the arterial, muscular, capillary, absorbent and secreting systems. If we had met with anything in the way of disaster in the use of the bandage, in a great variety of cases, the difficulty might be easily resolved.—But its success has been uniform, its results always beneficial, its comforts of the most gratifying character, and its celerity of cure beyond that of any other means known to us.

It is not our intention to write a monograph on the application of the bandage. Our main object is to show its merits in comparison with the rules of Dr. Walker and M. Bauden, which are quoted in the November No. of the

---

* John Bell's Surgery, 1st volume, page 122, quarto edition, published at Edinburgh in 1801.

New York Journal of Medicine, by Dr. Stone. In the course of our remarks, incidental branches of the subject may extend the inquiry beyond those points.

In the article referred to, Dr. Stone enters into an examination of the statistics of primary and secondary amputations, and they are of a frightful character. It seems that "the mortality which followed upon wounds in the Astor Place riot," gave rise to much discussion upon *compound fractures* and their treatment. "Primary amputations have been looked upon with suspicion, and the advocates of this doctrine have been dismayed at the unfortunate termination of every case, it is believed, that was touched with the knife."

In these circumstances, an inquiry into the resources of surgery is an imperative duty, with a view of ascertaining the best means, sustained by the best statistics. Instead of pausing at the inquiry, whether primary or secondary amputations are best, it seems to us more surgical, and more philanthropic to inquire whether either is necessary. It is admitted on all hands, that the mortality after either of them is very great, and we think it would not be materially increased by the abandonment of amputation in many of the cases where it is used. We are glad to see that Malgaigne, in France, has announced his opposition to the practice. His experience was of a character to make him look narrowly into the subject. In a campaign in Poland, in an army of 80,000 men, neither he nor his colleagues succeeded in saving a case after amputation of the lower extremities, and statistics, gathered from the Parisian Hospitals, from 1836 to 1842, show that the mortality in all amputations from wounds was about 75 per cent.*

There are other statistics, furnished by Dr. Stone,

---

* Dr. Stone's paper.

which demand attention. In the Massachusetts General Hospital, from 1821 to 1845, there were 367 fractures.—There were 33 cases of compound fractures of both bones of the leg; of these, 14 died—a mortality of 42½ per cent. Of the 33 cases, 10 were amputated; 7 of them died—a mortality of 70 per cent. In these statistics we find reference made to the suppurative period, a fact, for which we shall have some use in another part of this paper.

The same hospital furnishes 12 cases of compound fracture of the thigh, some of which were comminuted. Of the 12, 7 died—a mortality of 58⅓ per cent. In injuries of this kind, Baron Percy has calculated that not more than one-fifth recover. Mr. Guthrie thinks that not more than one-sixth recover so as to have useful limbs, and that two-thirds of his cases died, whether amputation was performed or not, and that the limbs of the other one-sixth were useless and painful. In Holland, in 1814, out of eight of these severe accidents not amputated, one only recovered, and this with a useless limb.—According to Mr. Hennen, not one has done well where amputation was deferred, and, when performed, two out of three died.*

The intelligent writer, from whom we have quoted so extensively, says: "The mortality in bad compound fractures of the thigh is great, and the fear of future evils and death has, no doubt, induced surgeons to adopt the military rule, and to operate at once. But yet, according to the expositors of the rule, amputation does not lessen the mortality." Dr. S. adds: "The late Astor Place riot has added to our experience in gun-shot wounds, and the uniformity with which death followed amputation, has attracted general attention, and led us to inquire if there

---

* Dr. Stone's paper.

might be an alternative, and whether a better fortune might not have attended a different treatment. We have seen three compound fractures of the thigh, from the riot, and since then a gun-shot wound of the knee-joint. All were amputated, in the New York Hospital, and all died."

These are instructive facts, and to those acquainted with the value of the bandage they are quite as singular as they are instructive. We have been so long familiar with uniform recoveries from these severe injuries, without any impairment of the limbs, that a large per centage of death in such cases is surprising. We have seen compound comminuted fractures of the femur, of the tibia, fibula, forearm and humerus invariably recover under the influence of the bandage. We have seen the most shocking injuries to the joints recover perfectly, and we are more at a loss to say what the bandage will not do, than to record its merits.

After the record of the results of authoritative and fashionable surgery, of which we have given but a mere sketch, Dr. Stone says: "Cases of recovery have now become so numerous, that all doubt in respect to their curability is removed, and a hope is indulged that a clearer insight into their peculiarities and treatment may render their mortality much less than that from amputation." The fruition of such hopes is desirable, and in order to contribute something to this salutary result, we have undertaken the preparation of this paper. There can be no higher object among the honorable pursuits of man, than that which improves the means of alleviating human suffering, and which, tending to the abridgment of the evils of erroneous practice, results in saving not only limbs, but life itself. In the wide range of philanthropy, no more elevated pursuit could occupy the mind, than to bring to an end that disastrous practice which has been so lamentably fatal in the hands of the very magnates of surgical art. Let us pause a moment and look at the phenomena of compound fractures, treated by authority, and thus get an observation for a point of departure. Dr. Stone says: "In all cases of compound fractures, and especially those made by musket balls, the wounds in the soft parts are narrow and irregular. Pus accumulates, and is prevented from being freely discharged. It consequently dissects up the muscles, forms abscesses along the limb, or separates the soft parts from the bones, which may thence become necrosed. The inflammation, which precedes the secretion of matter, is deeply seated, and may become excessive from the sharp and irritating extremities of the broken bone, or from numerous spicula of comminuted bone irritating the surrounding muscles. The inflammation may be so great as, of itself, to destroy life; or it may extend to the veins and lymphatics. Excessive suppuration may follow, which may extinguish life or necessitate secondary amputation, or the patient may die from purulent absorption. These are the dangers, and in Paris the period of the greatest peril was found to be from the eighth to the fifteenth day."

These are the phenomena of the injuries we have been considering; the controversy is as to the best management of this state of things, and the choice is between amputation, and getting along with these processes in another way. But surely the surgeon may imagine, at least, an object of infinitely higher interest than either—that object is the prevention of the appearance of the phenomena mentioned by Dr. Stone, which we have quoted elsewhere. If that object can be attained, we have reached an improved surgery. The surgeons who flourished before the days of John Hunter, were universally in the habit of dressing stumps, after amputations, in such a way that exfoliation of the bone was an invariable attendant. That exfoliation was then considered a process of nature, but it was no such thing—it was a process of very bad surgery. Is it not likely that some of this old material lingers yet in the world? May not surgical art still hold on to evils, now considered unavoidable, just as the exfoliation of bone was formerly considered a necessary consequence of amputation? The universal judgment of the old surgeons once was that injuries of the scalp could not be repaired under many weeks —and they took precious good care to make their practice fulfil the judgment. When the French surgeons announced that their flap amputations procured an easy and perfect cure, and that adhesion often occurred in three days, O'Halleran, a surgeon of repute, went into a rage. "I wonld ask," he said, "of the most ignorant tyro in our profession, whether he ever saw or heard even, of a wound, though no more than an inch long, united in so short a time? These tales are told with more confidence than veracity; healing by inosculation, by the first intention, by immediate coalescence without suppuration, is merely chimerical, and opposite to the rules of nature." O'Halleran covered the face of his stumps with rolls of linen, and called the suppuration, he thus produced, the rule of nature! Have we not O'Hallerans yet in the world of surgery? Even in the midst of such pestilent errors as these, John Bell laid down the rule that should govern now, much more than it does. He knew the evils and the true method of avoiding them. He says: "Whether the wound be broad, in the form of a flap, or long and deep, or a penetrating wound, *there is much danger, lest the sides of such a wound be not kept in close contact;* in such wounds we lay long and flat compresses along the tract of the wound, keeping them firm with a broad or firm rolled bandage, *which both prevents collections of matter,* and brings the sides of the sore into contact." In our judgment the great secret of true surgery is developed in that paragraph of John Bell's. In the valuable truth enunciated there, we have the clue to an improved surgery, as far beyond that contended for by Dr. Stone, as he considers his beyond the practice of amputations. In the treatment of compound fractures, gun-shot wounds and injuries of that character, we would not exchange that "which prevents collections of matter," for everything that has been uttered in favor of other forms of treatment. The counter-openings, the pulling at spicula of bone, and the division of membranes and tendons, often resorted to by the surgery of the present day, seem to us a legacy of the past, when the universal practice was to make deep incisions in gun-shot wounds, and to apply actual cautery, and boiling turpentine and oil in order to kill the poison of the wound. The reputation of Ambrose Parè enabled him to rid surgery of this barbarous course, but he did it, not by an enlightened appeal to the judgment of his times, not by the inculcation of sounder principles, nor through a better understanding of the nature of the subject, but having purchased a secret from a surgeon of Turin, of great celebrity in the treatment of gun-shot wounds, Parè made it known, and brought it into vogue. That secret was the manufacture of "the oil of whelps," made by taking a pound of earth-worms, and infusing them in white wine, and boiling two dogs in oil till the flesh separated from the bones and mixing the oil and wine. A mild ointment was thus procured, and it enjoyed a great celebrity in the cure of gun-shot wounds. This was certainly a great improvement upon the barbaric practices that were displaced by it—it at least kept the surgery of the day away from the wound and enabled nature to depend upon her own resources.—So far as the interests of humanity were concerned, there was great improvement, certainly, in transferring the boiling oil from men to dogs. The fancy of the times was that gun-shot wounds must have something to do with boiling oil, and probably no better plan could have been devised for removing its use from men, than the one that Parè adopted from conviction of its utility. From the introduction of gunpowder to the present time, it seems to us that the surgery of its wounds is on a par with Bishop South's description of the studies of the Apocalypse: he said, he never saw a man who did not go to the study of that portion of revelation, a fool, and return from it a madman. To many surgical writers, it seems to us that gun-shot wounds are yet an Apocalypse. In other things we may control the wild actions of disease; in this department we are taught only to aid them. If we have a serious paronychia, after the dead bone is removed, we may procure a new bone by maintaining the action of the parts, at the point of serous effusion or the deposit of fibrin, but if we permit suppuration to come on, we may not have new bone. This is true, too, of all those reparative processes where we depend mainly upon the soft parts for the reproduction of new bones. And if we apply the expectant treatment to compound fractures, we must look for that series of phenomena, described in the quotation we made from Dr. Stone; we may announce beforehand, extensive collections of pus dissecting the soft parts in various directions, and inflicting a mass of evil which the surgeon brings into the case, instead of preventing.

There is no agent as capable of closing the door upon that train of evils, now looked upon as an attendant, *ex necessitate*, of compound fractures, gun-shot wounds, and other contusions, as the bandage. It is worth all other means of cure ever commended to the attention of men, and we are firmly persuaded that the young surgeon who may purchase a bolt of common calico; tear it up into simple rollers, and practice the use of them upon the sexes, of various ages, and temperament, until the principles of their action are thoroughly learned, will be infinitely better armed for surgical success than if he could enjoy the privilege of fitting himself up with instrumentation in the establishments of Weiss and Hutchinson.

The bandage effectually controls the arterial and muscular systems, and, through them, all their subordinates. Fever, with ague, is not more effectually and certainly and safely controlled by quinine, than the capillaries, secretory and absorbent vessels, medullary tissue, and the osseous system are by the bandage, and when it is promptly resorted to, properly applied and attended to, the worst cases of compound fracture yield to its influence, and secure the safety of the patient, and the gratification of the surgeon. Under that treatment there is no difference between a simple and a compound fracture.

In bringing about union of the bones, we must be governed by the very same principles that guide us in procuring union of the soft parts—we must regulate the inflammatory action so as to bring it within healthy limits.

The material processes in the union of the osseous tissue and the soft parts are the same. According to Bordenave's experiments and Liston's conclusive statements, there is an increased vascularity in the vessels of the osseous tissue, for the extra duty that is to be performed, just as there is in an incised or contused wound of the soft parts. It is for the surgeon to say whether that increased vascular action shall range within the process of primary adhesion, or pass into a suppurative action, and from that, into still more serious complications.

There is one fact of immense importance, that cannot be too forcibly impressed upon the mind of the surgeon: we have already referred to it in our review of Stanley on "Diseases of the Bones," and we then promised to recur to it in our remarks on the bandage. The accurate observer referred to, in speaking of compound fractures, remarks that *suppuration frequently occurs in the medullary tube of the fractured bone*, and fistulous abscesses are then, of course, unavoidable. That this is an attendant only upon compound fractures, that are treated improperly, we have the best reasons for believing; we have seen the severest forms of compound fracture repeatedly, and we know of many that we did not see, but which were managed by the bandage, and we have never yet known an untoward symptom in these cases. The whole of that series of disastrous phenomena described by Dr. Stone, and which may be found in nearly all descriptions of these injuries, vanishes under the use of the roller. Under the influence of that simple agent, collections of pus, fistulous canals careering wildly among the soft parts, excessive febrile excitement, inordinate pain, secondary hemorrhage, shortening of the limb, gangrene, and all kindred appearances are absent. These and all questions about extension and counter-extension in these cases, which have been the *animus* of so much surgical controversy, may be quietly buried in the folds of a bandage. The fact thus announced is as certain as that announced by the French surgeons when they resorted to proper methods to secure adhesion in their flap-operations; the contrary will be found, only, by those who, as O'Halleran did, in the question of adhesion, resort to a plan of treatment that sustains their views.

Under the use of the bandage, at the beginning of the treatment, no openings are necessary for gleets, sloughs, &c., for the governor of a steam engine does not more quietly, effectually and certainly control all the movements of the mighty power committed to its keeping, than the bandage does the movements of the absorbents in removing all extraneous matters of this kind. The terminations in compound fractures, of which we hear so much in surgical works,—lymph and moderate suppuration, inordinate suppuration, abscesses, collections of matter and gangrene, from which the suffering and drain are so great as to cause the loss of limb or life—are unknown to the bandage treatment. Let us, however, illustrate the truth, for facts are the best tests of reasoning.

G. B. Didlake, Esq., of this city, a gentleman about 57 years of age, had been the victim to disease of the tibia of the right leg for some time. In addition to this, he was for many years the subject of varicose veins of a formidable character, in the same limb, by which he was frequently confined to the house for several weeks. Last year, while engaged in a fracas with a large, stout young man, he received a kick which broke the bones of the right leg; drove the end of the tibia through the soft parts into his sock, and produced hemorrhage. A messenger was immediately despatched for his surgeon, Dr. Flint, but failing to find him, we were requested to take charge of the case. A more forbidding one could scarcely be found : a compound fracture, complicated with age, numerous spicula of bone grating like egg-shells, the patient debilitated by a previous derangement of the fractured bone, and by a serious condition of the veins, absorbents and secretory vessels of the leg. Long experience in the use of the bandage, and observation of its salutary powers in the most forbidding circumstances, however, induced a reliance upon it. With such assistance as we could command among the bystanders, we succeeded in adjusting the bones, and dressed the fracture with a simple roller, passing from the toes to the knee, in such a way as to command the parts that were to perform the duty of reparation, and to respect the complications of the case. After the application of the bandage, pieces of patent felt, which had been cut to suit the limb, were dipped in hot water, and, while soft and pliable from the heat, were moulded to the limb, and securely fixed with the roller. One of the good qualities of this "felt" is, that while heated with hot water it may be made to adapt itself as perfectly to a limb as plaster of Paris does to a mould, and, when it cools, it is a perfect splint.

Soon after the limb was dressed, we met Dr. Flint; and at his request, saw the patient some days with him. Mr. D. suffered no pain from the fracture, but often expressed his comfort. The dressing was not disturbed until there was assurance that the bones were uniting; and was taken off then, to be adjusted to the altered condition of the limb. In three months, the patient was going about, and he walks now better than he generally did before the accident. Under the genial influence and supporting power of the bandage, and a careful system of diet, this limb, with its serious complications, was not only saved, but improved; what would have been its fate under any other treatment, let the records of surgery testify. We shall look into some of these records presently.

In 1847, a young man named Lefferson Dye, during a fire on Walnut street, rolled from the roof of a house three stories high, and fell on a brick pavement. One ankle joint was very much crushed, and the malleolii were fractured and comminuted. Having prepared the roller and patent felt for the case, we requested Dr. Flint's assistance in adjusting the fractures, which he kindly gave. The limb was dressed with the bandage, and the felt was used as splints; in a short time the suffering, which had been excruciating before the dressing, ceased, and the limb was dressed but twice afterwards.—At the end of two weeks, we met the young man on crutches, the foot being held from the ground, on his way to a meeting of his fire company; and in less than a month from the time of the accident, he resumed his labors in the foundery, where he was serving an apprenticeship. We are informed that he has a limp in his gait which is scarcely perceptible, but he might have avoided even that, by proper attention to the medical instructions given during his convalescence.

Early in July, 1847, a young man named Jarboe, while nailing laths near the garret of a house, three stories high, missed an object at which he was catching, and was precipitated from his scaffold, through the window, to the ground. He fell on a small conical mound of brick and rubbish, which had been trampled until it was almost as hard as a mass of brick. The young man was picked up immediately, and conveyed into the house, and the supposition was that he was dead. We were immediately sent for, and upon reaching the house, considered his case hopeless. By the use of proper applications, however, he slowly revived, and, upon examination, we found a compound, comminuted fracture of the humerus. The parts were adjusted as perfectly as possible, the fingers were separately dressed with a narrow roller, and the hand and arm were then enveloped in a bandage; the integrity of the adjustment was guarded by the patent felt. The boy was then conveyed to the house of his instructor, and the threatening reaction was controlled by the lancet and purgatives. He complained but little of his arm, after the bandage was applied; the circulation was very sluggish for about twenty-four hours, but gradually came up to a healthy condition. In less than two weeks, he was walking the streets, and within a month was using his arm. He is now one of the quickest and best hands, of his age, in the city, and has as perfect use of his arm as he ever had.

A daughter of Mr. Thurman's, a beautiful child, about four years of age, while playing among the frames of a building, in 1849, was caught under a very large and heavy door frame, which fell with its whole weight upon her thigh, and crushed the femur. We reached the case in a few minutes after the accident, and applied the bandage and patent felt to this serious fracture. The sufferings of the child were agonizing, until the bandage was applied. This relieved her in a few minutes, and she recovered in three weeks, without any inconvenience.

About the time of her recovery, two small boys, on the same day, in different parts of the city, had their arms fractured; in one of them there was fracture of the condyle of the humerus; in the other, there was fracture of the head of the radius. The bandage was applied to these boys, and in three weeks they were perfectly well, with a single dressing of their arms.

On the day following the accident of the boys, a little girl, about three years old, a daughter of Henry Wolford, city clerk, fell from a horse and fractured the femur. We saw her soon after the accident, and applied the bandage, and she recovered in three weeks, without a troublesome symptom.

We might go on, filling the pages of this Journal with cases of this kind, but we deem it unnecessary.

In the cases we have reported, of severe injury, we have that state of things described by Dr. Stone. He says: "In civil life, *most of the accidents that necessitate* amputation are caused by lofty falls," or by other accidents "which not only break bones but produce general contusions to a greater degree than is likely to be met with in gun-shot wounds." Two of the cases we have named fulfil the conditions that "necessitate amputation."

The young fireman fell from the roof of a house three stories high, and not only fractured, but crushed the bones of his ankle joint, and of course had severe contusion; the young plasterer fell from the attic of one of the tallest houses in Louisville, and produced a very serious fracture. The limbs were saved, were not shortened, and were restored to perfect use, with but little suffering on the part of the patients, and with scarcely any confinement. If arterial action had been suffered to run wild in these cases, and no controlling power had been used over muscular irritability, and the absorbent and secretory systems, either the limbs or lives, and probably both, of these youths would have been lost. As it is, they are as well, in all respects, as before the accidents that befel them.

But for the support of the bandage, the gentleman whose case we first detailed, would have left his leg to some anatomical museum, to prove to another age, how apt necrosis and gangrene are to supervene upon compound fracture, where the complications we have mentioned were present. A diseased bone, fractured and driven through the soft parts, the veins in a varicose state for many years in an aged gentleman, were serious circumstances, and if the vascular parts had been permitted to swell and inflame, would have presented suppuration of the medullary tissue of the bone, exfoliation would have followed, then necrosis, and gangrene of the soft parts would have demanded amputation. But the bandage not only saved this limb, but recuperated it. The cases of the children are reported to show how perfectly the bandage is adapted to their condition, but it must be remembered that children cannot bear a very tight bandage.

But we have, what others may think, cases of more gravity than these—compound fractures of the thigh.—These are considered among the most formidable compound fractures that are curable, and they, under the ordinary treatment, most frequently require amputation.—We could give abuudant details of cases of this description, but there is no good reason for lumbering up this paper with reports of a multitude of cases of this kind, presenting a mere repetition of the same matter in a great variety of forms. It is enough that we illustrate the power of the bandage in this kind of injury. In thus restricting ourself to two cases, it may be proper to say that we know of many others, and that we never have known one, treated by the bandage in the hands of one who understood its use, that did not fare as well as the cases we now give. We make an abstract of one of the cases, from a very full report of its phenomena, made by T. L. Caldwell, M. D., of this city. This report was published in 1838.

A young man, eighteen or nineteen years of age, was admitted into the Louisville Marine Hospital, on the morning of the 10th of September, 1837, with a compound fracture of the left thigh. In consequence of some misdemeanor the preceding night, the police officers were in pursuit of him, and when the pursuit closely pressed him, he sprang into a pit about twelve or fourteen feet in depth; the compound fracture was the result of the jump. He was taken from the pit, and left lying upon the sidewalk until daylight; was then carried to jail; remained there some hours, and was then conveyed on a dray to the hospital. Considerable reaction had taken place; the suffering was great; the limb considerably curved at the point of fracture, and the surrounding soft parts much swollen. The external wound was about an inch in length, on the outer side, about four or five inches below the trochanter major, made by the end of the upper portion of the fractured bone, which had torn its way through the mass of muscular fibre, forming the vastus externus. The fracture of the bone was oblique, running upward and inward in the direction of the trochanter minor. The patient was well set and muscular, was irregular in his habits, but had not impaired his constitution.—There seemed to be but little hemorrhage.

After having the limb washed with clean water, Dr. Caldwell dressed it from the toes to just above the patella, with a common roller bandage. Extension and counter-extension pullies were then applied for the purpose of bringing the fractured ends of the bone to their proper position, and by the aid of bleeding, this was soon accomplished. As soon as the ends of the bone were in apposition, another roller bandage was continued firmly from the extending point to the groin, and one or two turns were taken around the body, to keep the bandage from slipping. The external wound was merely covered with a piece of dry lint beneath the bandage. The pullies were then removed, and the space above the knee, which had been occupied by the extending bands, was covered by a short roller, which completed the envelopment of the limb. The patient was then placed on a mattrass, prepared as a common fracture bed, by which he was kept perfectly still. He was occasionally purged by mercurial cathartics, from the 11th of September to the 17th. On the 15th he was attacked with diarrhea, which readily yielded to one dose of laudanum. Throughout all this time, the patient was free from pain in the limb. On the 17th the dressings were removed for the first time; there was neither swelling nor inflammation in the limb; its form was natural, and without the least shortening, "*the action of the muscles having been completely and as effectually controlled by the bandage alone, as could have been done by the most complicated fracture apparatus for keeping up extension.*" The wound through the soft parts was closed by the first intention, and only a small circular spot, about the size of a six and a fourth cent piece, was not covered by skin—it showed where the bone had protruded. No sinus existed, nor was Dr. Caldwell able to press any matter from the wound. He correctly remarks, that the case might now fairly be considered as reduced to a simple fracture—a fact of inestimable value, upon which we shall insist in another part of this paper.

On the 17th, the dressings were renewed, care being taken to prevent displacement, and the bandage was found to maintain such a condition of the parts, that it was only necessary to support the foot to prevent any unnatural torsion of the limb.

From this time to the 3d of October, every thing went on well with this case. The limb was comfortable, sleep composed, and the natural functions properly performed. On the 3d of October, the limb was dressed for the fourth time, and in the night a severe diarrhea again seized the patient. This was soon relieved. It was ascertained that union was slowly taking place, the obliquity of the fracture interfering, of course, with celerity in this process. The patient, too, tried experiments with the limb, frequently, and Dr. Caldwell's attentions ceased in the first week of November. The patient was making experiments in the night, unbeknown to his surgeon, to ascertain when his limb would be strong enough to enable him to elope from the officers of justice, and this helped to protract his case. But on the 16th of December, close examination showed that bony union had been effected, and early in January the patient made his escape from the hospital.

We believe that we have given the main incidents of this interesting case. It was managed with skill, and terminated in an admirable success. We repeat that we give these cases as illustrations of the bandage treatment —it would not be difficult to fill this number of the Journal with reports of cases of fracture, that we have treated with the simple roller, and we have never seen an untoward symptom, as a consequence of the treatment, nor but one unfortunate termination of a case of fracture thus treated.

We submit another case of severe fracture of the femur, the details of which were furnished us by the surgeon who treated the case. The severity of the injury caused a great wonderment at the time, over the recovery of the patient, and knowing its history, we requested of the gentleman who had charge of the case, a report of the treatment. We regret that he will not permit the use of his name. He reports:

"Hiram Jones, a painter, fell from the top of the hospital, three stories high, while painting, by the turning of a ladder, on the top of which he was at work. It fell across another large ladder, lying on the ground, with the side of which Jones's head and left side came in contact, producing a terrible concussion of the brain and a comminuted fracture of the upper portion of the femur—the fragments of the trochanter being distinctly felt and recognized by the crepitus.

"He was at once carried into the hospital, and the surgeon sent for reached him in about fifteen or twenty minutes after the accident. The urgent symptoms of the injury to the brain first calling for the chief attention, only a temporary dressing was applied to the injured limb.—This was a roller bandage, commenced at the toes and carefully applied with moderate pressure, so as to control muscular action, and continued up so as to cover and control the glutei muscles. There was little hope, at the time, of his surviving his injuries, so serious were the symptoms. By prompt and energetic treatment, the cerebral symptoms were, however, successfully combatted, and about the fifth day, it was deemed proper to adopt such treatment for the injured limb as gave the best promise of success. When the bandage was removed the limb by the contraction of the muscles became shortened, so as to lead to the inference that the fracture involved the neck of the femur. By steady and gentle traction it was brought to its proper length, and again a roller was carefully applied, with somewhat increased power, to the entire limb as before, carefully controlling the glutei muscles. Over this dressing, to the outer and posterior portion of the limb, from the heel to near the crest of the ileum, a splint of thick trunk board, cut to suit the form of the outer part of the limb, and softened by hot water, and another board to suit the inside of the limb, from the groin to the knee, so as nearly to meet the former, were placed upon the limb. These, in their soft state, were made to apply closely to the bandaged limb, by the firm application of another roller bandage over the whole extent, thus converting the dressing into something analogous to the 'appariel immobile' of the French. The patient suffered little or no pain now, and the cerebral symptoms gradually subsiding, the limb became the more important object of attention. The first dressing, as described, remained undisturbed till about the tenth day, when it was deemed proper to remove and re-adjust it. The splints were perfectly moulded to the form of the part. All swelling seemed to have subsided, and the limb continued of its proper length, but as yet there was no appearance of course, of real union in the fractured portion. The entire dressing was reapplied without moistening the splints. Everything went on well with the patient, and about the ninth day after, the dressing was applied the third time. At this examination partial union was found to have commenced, there being little or no crepitation in the fractured portion. The dressing was re-applied as before, and again allowed to remain till about the tenth day, when it was removed, and to the astonishment of the attending surgeon, on testing the limb by twisting and turning it in various ways, no evidence of fracture or weakness of the injured portion could be detected. The dressing was not re-applied—there was, of course, temporary stiffness from the long confinement of the muscles, and some stiffness of the knee joint. Friction with the hand, and some stimulating ointment were directed and the patient ordered to remain strictly quiet till crutches could be prepared for him and permission should be given him to rise. There was not the least shortening of the limb. In a very few days, less than a week, he was put on the crutches, and began to move freely about with them. He left the hospital and was so imprudent as to lay aside his crutches and resort to a cane prematurely, and before the cartilaginous union had fairly consolidated. The consequence of this was a slight outward curvature of the limb, occasioning a shortening of it from a half to three-quarters of an inch. He is now an active mechanic, industriously pursuing his business, which he recommenced a few weeks after leaving the hospital."

It is not necessary to dwell upon the skilful use of the roller, and the extraordinary success of its application in this case. Its merits speak for themselves.

We know quite a large number of gentlemen skilled in the use of the bandage, who are often called upon to use it, who rely upon its certain success with as much security as they do upon quinine in arresting an ague, and from this enlarged experience we have the most gratifying assurances of the vast superiority of the roller treatment, over every other form of treatment that has ever been suggested for fractures of every description. It is a misfortune to humanity that an understanding of the merits and application of the roller, is not as universal as the demand for its use. It is painful to read the records of surgery, military surgery especially, even in the hands of such men as Guthrie, Larrey, and Hennen—painful enough from the evils strewed along the path of reading; and more painful from an absolute knowledge that a better surgery would have materially lessened the evils.—The voice of Mr. Pott is yet potential among surgeons. At this moment France is giving terrible evidence that some other surgery than Mr. Pott's must be attempted, and there the surgery of compound fractures turns to the forlorn consolation that nothing unattempted could be more fatal than what is done. It is not at all wonderful that Mr. Pott is reverenced, for he is richly entitled to the respect and esteem of his professional brethren, but inasmuch as a medical man has no right to love anything, even his own reputation, better than the welfare of his patients, why should he reverence anything that stands in the way of that welfare? Hear Mr. Pott now on the subject of compound fractures, and remember that his rules govern in surgery now: "In a compound fracture, the first object of consideration is, whether the preservation of the fractured limb can, with safety to the patient's life, be attempted; or, in other words, whether the probable chance of destruction, from the nature and circumstances of the accident, is not greater than it would be from the operation of amputation. Many things may occur to make this the case. The bone or bones being broken into many different pieces, and that for a considerable extent, as happens from broad wheels or other heavy bodies of large surface, passing over or falling on such limbs; the skin, muscles, tendons, &c., being so torn, lacerated and destroyed, as to render gangrene and mortification the most probable and most immediate consequence; the extremities of the bones forming a joint being crushed, or as it were, comminuted, and the ligaments connecting such bones torn and spoiled, are, among others, sufficient reasons for proposing and for performing immediate amputation." This is the rule of modern surgical practice, and as such we must examine it. In our remarks on Mr. Stanley's work on "Diseases of the Bones," we referred to his singular diagnosis in some cases—singular in being based on the treatment; one set of remedies was to be tried—if it failed, the disease was not the one to which that set was applicable. It strikes us that Mr. Pott's decision in compound fractures, has a basis akin to it; a certain set of symptoms springs up, not, as Mr. Pott thought, in spite of the treatment, but as an inevitable consequence of it, and upon these evils he founds another disastrous series. He did not pause to enquire patiently of nature, whether he might not prevent all the untoward symptoms he describes as leading to immediate amputation. Mr. Pott, unfortunately for himself, and for the science and art of which he was a distinguished ornament, was much more skilful in the use of amputating instruments than of the bandage. If he had understood the philosophy of the latter, he would have enabled Mr. Samuel Cooper to say, that certain forms of dressing will convert a compound into a simple fracture, instead of saying they almost do it.

What are the demands of fractures? If a surgeon permits the lips of an incised wound to stand apart, there can be no union by primary adhesion, but suppuration, and, perhaps, ulceration will follow. If he dresses the wound so imperfectly as to make the adhesion the victim to many chances, the rule is still uncertain, and we may have an M. Roux telling us that surgeons are sometimes lucky and sometimes unlucky in the same line of cases. But if the wound be dressed so as to maintain coaptation and perfect rest, we have none of that variety of result that attends uncertain dressings. The same doctrine is true in fractures of the bones; if they are dressed so as to make it a matter of uncertainty whether the ends of bone remain in proper position, we have uncertainty of result, but if the dressing secures the object perfectly, and keeps the inflammatory action true to union by the first intention, we have uniformity of result. It is of the greatest importance to keep the ends of fractured bones motionless; a callus is in fractured bone, what a cicatrix is in an incised wound, and each may be obtained in the same way. Of all the causes of displacement of the ends of fractured bone, there is none to be dreaded as much as the action of the muscles. Their origin may be above the point of fracture, and their insertion in the lower fragment; consequently we have most difficulty to apprehend from the lower fragment. All writers acknowledge the little control that splints exercise over the short fragment of bone near the articular end. A bone broken in several places, and an oblique fracture present difficulties in preventing retraction of the short portion. The difficulties we have named belong equally to simple and compound fractures; in the latter we generally find the following additional evils: hemorrhage, violent and excessive inflammation, extreme pain, large abscesses, delirium and fever, and gangrene. Of the difficulties we have named, any one may satisfy himself by looking over the vast variety of apparatus for fractures. In proportion to the fatality of a disease is the number of remedies for it, that never fail. In cholera we have an innumerable multitude of medicines that cured every case! The same state of things belongs to the apparatus for fractures; each kind is great in its way, is an improvement upon all others—but the difficulties remain. Hence the variety of apparatus.

The philosophy of the bandage shows how eminently that agent is adapted to fractures. In its use, instead of having to combat that irritability of the muscular system which all writers acknowlege is a difficulty of magnitude, in other forms of management, the bandage makes the muscular system the main agent for preventing retraction of the broken bone. Bone can only move in obedience to the muscles, and if they are immovable, the broken bone will be immovable. No contrivance for keeping the ends of the bones together and maintaining them in their place, can be compared to the mass of muscles in the limb, in a state of perfect quietude. When the ends of the bone are brought to their place, and a firm bandage, pressing equably and properly from the extremities of the limb to the proper point above the seat of fracture, is placed upon the muscles of the limb, they are as incapable of motion as the bones. The muscles, therefore, instead of being a difficulty in the treatment of fractures become an important element for success in their management with the roller, and if they are properly used, we not only secure union of the bone, but integrity of the length of the limb; and we need feel no kind of concern about those questions that have puzzled the best of surgeons—the apparatus for extension and counter-extension, the frequency of its use during the cure, &c., &c. On the subject of the roller, Mr. Guthrie says, a bandage is harmful because it prevents swelling, and a certain amount of that is useful to some extent. We grant the latter part of the statement, it is the *uncertain* amount of swelling against which the surgeon should be on his guard, and for this purpose the roller is worth all other means.

Dr. Caldwell's case is but one of a multitude of similar ones that illustrate, very forcibly, the fact that by the use of the roller, in compound fractures, those formidable injuries are reduced to the character of simple fractures.—And it should never be forgotten, that when the joints are involved, or endangered in fractures, that the ordinary treatment utterly fails, while the roller offers a guaranty of safety that may be sought in vain, in any other resource of surgery ever offered to the world. The remarks of John Bell on the inestimable advantages of the roller, in the treatment of diseased bursa, are as applicable to these cases as to those specially indicated by him. C. Wilson Cruttwell reports cases of diseased joints, in which, having exhausted all the ordinary means of cure in vogue, he was driven to the roller treatment, and succeeded with it. Richerand and Brodie amply confirm the value of the roller in such cases. Mr. Scott obtained great celebrity in the cure of joints, by a bungling kind of shingling of soap cerate, emplast. plumbii., mercurial ointment, and strips of leather. In connection with this stratified treatment, he used the roller, and to that his cures belong, for we have seen cures made with the roller alone. The value of the bandage in fractures involving the joints cannot be disputed, hence the claim we make for it—while it accomplishes all the good that can be gained by any other plan, it has results of its own, that belong to no other treatment. And whether a fracture is intra or extra-capsular, the bandage is the best treatment, and with its proper use, the surgeon may as certainly look for osseous union in a case of intra-capsular fracture, as when the fracture is external to the capsular ligament. Mr. B. Cooper has recently undertaken to sustain the views of his uncle, who believed that intra-capsular fractures did not unite by osseous matter; but that they do is sustained by too many facts to be shaken.

But to resume; if the ends of the bone are placed in their proper position, if they are maintained there by a dressing that destroys all power in the muscles for motion, common sense teaches us that the ends of the bone in compound fracture will unite just as they do in simple fracture, and while this process is going on in the osseous system, the effects of the contusion or laceration of the soft parts will, under the influence of the roller, recover and get well long before union has taken place in the bones. These are the teachings of common sense, and are confirmed by a great number of cases in practice. In the treatment of compound fractures used by Desault, Boyer, Stromeyer, Vidal, Amesbury, and a host of others, the surgeon must expect the soft parts to undertake some method of cure, while the reparative process is going on in the ends of the bone; with the means in use he looks for suppuration, granulations, &c., with as much certainty as he looks for union in the bones, while the surgeon who uses the roller, is certain that his case will not suppurate, and that he will procure union by the first intention, in the soft parts as well as in the fractured bone. This is no fancy sketch, no statement of a controversial nature, of the operations of common apparatus in fractures; but the sad and sober truth, gathered from surgical records. In order to make the matter impressive, let us gather the fruits of experience from hospital reports: a narrow inspection of them may lead men to acquaint themselves with the roller, and give the world the benefit of that improved knowledge. We make our summary from the reports of London hospitals, and take them as they have risen to view in our reading. Let the reader imagine, when he sees the stereotyped phrase, "the usual means," that he sees men stretched on what seems to be the debris of a Spanish Inquisition, and that he stands in the very presence of instruments of torture; in the presence of the rack itself, and he may aid himself in forming a just conception of the scene. The apparatus of Mr. Bottomley is supposed to be the perfection of fracture machinery, but whenever we look at it in drawings, we instictively search the picture for the Inquisitor General.

W. F. Vidal, M. R. C. S. and House Surgeon to the London Hospital reports a case of compound comminuted fracture of the right leg, attended with nothing like the serious complications of G. B. Didlake's case, reported in a preceding part of this paper. The fracture was put up in splints in "the usual manner." The hemorrhage, at first slight, increased; the wound was enlarged, a loose piece of bone removed, and "the soft parts being extensively lacerated, amputation was determined upon." Compare this result with the roller treatment in the case of G. B. Didlake.

In the Royal Free Hospital, T. Wakley reports the case of a man, sixty years of age, who was thrown, on the 17th of June, from a cart, and produced fracture of the fibula, dislocation of the foot backwards, with a consecutive fracture of the inner malleolus, and dislocation of the tibia forwards. A case more eminently requiring the roller could scarcely be imagined. The fractures and dislocations were reduced, and the limb placed in a splint. The patient complained of much pain from cramps in the injured limb. The limb swelled greatly on the 19th. July 2nd, the patient had a rigor lasting half an hour. The skin was sloughy in places, and fluctuation was evident around the joint. July 3d, absorbent inflammation up the thigh. 6th, an opening was made, which discharged three or four ounces of very fetid pus, mingled with synovia —slough extending. 7th and 8th, great discharges of pus, and sloughs increasing. On the 12th the limb was amputated, and on the 20th the man died. The autopsy showed that the fibula was broken in two places, the inner malleolus torn off, the joint in process of destruction —the whole of the soft tissues of the limb were sloughy and disorganized.*

This is the natural result of suffering inflammation to pursue its own course. This patient should have done as well as the fireman of the Washington Fire Company, whose case we have reported, and under the use of the roller the recovery would have been certain.

In Bartholomew's Hospital Reports, we have the case of a compound fracture and dislocation of the bones of the wrist joint, from a fall of eight feet, on the 11th of December. Mr. Lawrence treated the case in "the usual way." On the 18th, extensive discharges of pus were procured; sloughs showed themselves, delirium came on, and on the 22d death took place.†

At a meeting of the Royal Medical and Chirurgical Society, April 13th, 1847, Mr. Partridge reported a case of comminuted fracture of the radius, "treated," to use his own words, "on the usual principles, but in a few days after the accident he came into the hospital, *abscesses having formed*, and pieces of bone having come away," and amputation had to be performed. This statement caused no kind of surprize among the gentlemen present, but among surgeons acquainted with the roller, it must excite the greatest surprize, to see cases of this kind demand amputation.

At the same meeting, Mr. Norton Shaw, the translator of Professor Fenger's work on fractures of the radius, said: "Acknowledging, then, the frequency of this fracture, [of the radius,] and the repeated failures attending its treatment, (Velpean cures but thirty out of fifty,) thus

---

* London Lancet, Oct., 1848, p. 335. † Ibid., Nov., 1847, p. 410.

causing a partial, if not total, loss of the use of the hand and fingers, it will be readily conceded than any rational plan, by which a more favorable result may be attained, is worthy the notice of the profession,"* and the rational plan suggested is a splint, that will support the arm, and let the hand hang down! We are really surprized that any surgeon should experience difficulty in these cases: we have never met with anything of the kind, with the roller treatment, and we have seen a large number of simple, and some few compound and comminuted fractures of the radius. We never saw one fail to do well, and never had any suppurative action in such cases. Nor can we imagine why there should be any more difficulty in the treatment of a fractured radius, than of any other bone. It may not be amiss to mention here, that in treating fractures of the radius or ulna with the roller, it is necessary to make semicircular splints, to apply to the outer and inner parts of the arm, the full length of the bones, in order to press down between the bones, to keep them apart. Without this precaution, the treatment of the case will fail. Rolls of paper will do very well, instead of the small splints of wood.

We have, now, gathered the light of the times upon the common management of fractures of the bones of the arm and leg, and have found that "common management," far behind practical medicine, and very far behind surgical science in almost all its other departments. Everything in the examinations we have made warns us that something must be done to remove that deplorable waste of life, the mournful destruction of limbs, and the agonizing sufferings of patients from fractures, which meet us almost everywhere in the surgical records of the times. In the treatment of fractures men move in the beaten

* London Lancet, August, 1847.

path of a circle; all their movements are motions on the periphery of that circle, and few seek to make a departure from it. But it is time that something should be done, and no matter what that may be, it is problematical whether anything could be much worse than the present state of surgery in severe cases of fracture. The testimony of surgeons of the highest repute, shows how constantly, in compound fractures, the amputating knife is resorted to, either primarily or secondarily. We see Roux and Malgaigne in opposition on the question of amputation—one advocating primary, the other urging that an effort may be made to save limbs, without making amputation any more dangerous than it now is, even if it should be the ultimate resort. We have presented the humiliating testimony of Guthrie and Hennen, on the resources of surgery in severe cases of fracture; we have quoted from Dr. Stone the results of the surgery of the Astor Place riot, and have found them almost as disastrous as the fusilade of the military, and we have wandered into the great hospitals of the world, and surveyed surgery, in its practical results, in these severe injuries. And we repeat the impressive lesson, that there is not a feature of the common management of fracture, that does not plead trumpet-tongued for a great change in the treatment. There is not a principle involved in fractures that does not as plainly point to the roller treatment, as calculous disease points to lithotomy. Under that treatment, injuries that are formidable with all other management, become as tractable as ague is under quinine administration, and limbs are saved and restored to perfect use, that would be amputated in nearly all the great hospitals, or by nearly all the great surgeons, in the world. It is not a little surprising to find the surgery of the West and South, in these cases, so infinitely in advance of the great centers of medical and surgical sciençe. What is there considered formidable, we, in the West, in possession of a simple, philosophical and natural treatment, find tractable and manageable. The disasters of their surgery are unknown in the West and South. After practicing for years in compound and comminuted fractures, gun-shot wounds, injuries of the joints, &c., with the roller treatment, the pathology of similar cases, reported under the best European treatment, seems to belong to a different series of injuries. The hemorrhages, caries, abscesses, sloughs, fistulous canals, &c., that are common attendants upon fractures in Europe, are not merely modified, but are entirely unknown to a more enlightened surgery. In a case presenting a severe compound fracture of one leg, and a simple fracture of the other in the same individual, the chances are, that in European practice, one limb, at least, would be consigned to the amputating knife. But under the roller treatment, no difference would be recognized in the management of the two, and none would be found in the result. Both would get well alike; there would be no more suppuration in the compound, than in the simple fracture, and both limbs would recover at the same time. We have known this so frequently that we speak of the result with just as much certainty as we do of the curableness of ague with quinine. The roller treatment knows no difference between a compound and simple fracture. We exert an influence with it, upon the muscular, arterial, secretory, and absorbent systems, that is unknown and alien to any other treatment; we gain results that are never found in any other, and avoid inflicting sufferings and injuries that are the concomitants of all others. When fractures, gunshot wounds, &c., are treated by the roller, the pathology of such injuries presents such a distinct set of peculiar features, that it seems almost impossible that it can belong to the classes of cases under the same names treated by "the usual means."*

Having thus compared the two systems of treatment, and found the immeasurable difference there is between them, the difference between life and death, between freedom from pain and suffering, and the presence of the profoundest anguish—there remains but one more point in this subject, that requires scrutiny. Men who are wedded to the use of fracture apparatus, suffer an ill-founded prejudice to cloud their minds on the subject of extension and counter-extension, and we now purpose to look into this subject. We have referred to it several times in the course of these remarks; and have a fine illustration of the extension and counter-extension offices of the roller in the admirable case we have quoted from Dr. T. L. Caldwell, in the case of the painter, and in the cases of G. B. Didlake, the young fireman, and Mr. Jarboe. None of the innumerable contrivances, that have been invented, ever held broken bones to their place as admirably, as a well applied roller will universally do it. And it must be borne in mind that the best contrivances of the splint often fail in this essential point. This is admitted and deplored by all who use them. The greatest care is taken

---

* In the 2d volume of "The Transactions of the American Medical Association," the reader will find a reference to Prof. F. H. Hamilton's Fracture Tables, These tables embrace 136 cases of fracture, some thirty of which were supposed to be in persons who were able to command the best services, for treatment, in the country, The book referred to says: "The results are sufficiently humiliating, and will perhaps surprise even those who have not formed a high estimate of the resources of our art. For instance, only two cases of fracture of the shaft of the femur, out of fifteen, occurring in adults, have resulted without deformity. Of twenty two cases of fracture of the tibia and fibula, seventeen are imperfect. Of thirteen instances of fracture of both radius and ulna, six are imperfect. Of fourteen cases of fracture of the humerus, no less than nine exhibit imperfect results." And yet the attention of the American Medical Association was directed to splints, instead of the bandage! The prejudices of education, are almost almighty!

to find "the exact locality of the fracture, how many inches and lines it lies below the articulating head of the bone, and how far it has occurred below the insertions of the psoas and iliacus muscles,"* and after all this care the failure to derive benefit from it is often assured by the mechanical contrivances resorted to. Maclise says: "When the femur is fractured immediately below the insertion of the psoas and iliacus muscles, or even at the middle of its shaft, the fragments of the bone are drawn widely apart, owing chiefly to the traction of the muscles named above. The upper end of the bone is rotated almost perpendicularly to the horizontal position of the body, and the lower end, obeying the contractions of all the larger muscles of the thigh, is drawn up behind the upper fragment; if the fracture has occurred obliquely from behind, downwards and forwards, or from before, downwards and backwards, then the lower fragment is retracted. In either case, the plan of treatment indicated is to approximate both fragments, and fit their sundered extremities as closely to each other as possible, and to this end it is that extension and counter-extension of the limb are made." Modifications of Desault's long splint, or M'Intyre's, or the double inclined plane, are the usual resorts. Again, Maclise says: "The patient is lying on his back; we feel the distal end of the upper fragment of the femur pointing to the fore part of the thigh, indicating that the axis of this part makes a more or less acute angle with the axis of the lower fragment: we exercise no command over the position of the upper one;—how, therefore, can we expect that the horizontal extension, whatever be the degree to which this is made, can be the means whereby the upper fragment is to be forced into a

*Maclise's Remarks on Fractures.

line of axis with the lower? *Desault's splint does not effect this.*"

"Owing to the nature of the parts concerned in such fractures of the thigh bone, the deviation of the upper fragment from the line of axis of the lower, is masked to the surgeon, *and it is doubtful whether it be possible to bring both ends of the broken bone in strict apposition by any fashion of apparatus hitherto invented.* * * * If we see reason to praise the long splint of Desault, for its efficiency in maintaining extension, we cannot ascribe to it a fitness in bringing the fractured ends in contact; and, on the other hand, if we recognize the merits of the double inclined plane, in causing the lower fragment to coincide with the axis of the upper, by following this latter to its new position, we know that in the use of it, all power in maintaining the necessary extension is lost.—Though the fragments lie parallel, they are not placed end to end. In fractures of the upper third of the femur it actually occurs, that although, to the outward seeming, the thigh corresponds in length and calibre with that of the opposite and sound side, yet the two fragments of the bone may, at the same time, be drawn so far apart, as that the broken point of the upper one may be felt subcutaneous at the fore part of the limb, while that of the lower may rest deeply buried in the centre of the muscles; *and we have noticed such a condition of the parts to remain, even after the supposed reduction of the fracture,* and the application of Baron Boyer's screw splint," a modification of Desault's invention.

As a corollary to these statements, listen once more to Maclise: "At the Dupuytren Museum, in Paris, may be seen an interesting collection of fractured thigh bones, *united in every possible position which any two freely moving bodies can be supposed to assume. In this collection there is no example of exact co-aptation between the ends of both fragments.*" This is certainly a powerful plea for reform in the treatment of fractures, even if we had no other.—We have preferred the use of the remarks of Mr. Maclise, rather than our own, because he is an advocate for the use of splints, and his testimony to their utter worthlessness may have more effect than any evidence we might give. Abundant reason has been given already for the abandonment of what surgeons glibly call "the usual means," of treating fracture, but we must appeal once more to Maclise, on the subject of splints, and if any one, after examining the statement, chooses to rely on such apparatus, he can enjoy the satisfaction of sinning against light and knowledge. Maclise says: "If there were any well approved apparatus for fractures of the femur, one might expect that the use of such would be universally adopted; if this were the only one which could meet all contingencies, it is certain that no surgeon acquainted with its value would, (following the conceit of his own originality) prefer another and less efficient apparatus, even though he himself were the inventor of the same. *But there is no such apparatus, and hence it is that in each of the great hospitals of Paris, we see various modes of treating these fractures*," The splint is a part of each of these various modes.

It is seldom that such an array of truth can be brought to bear against a long established custom, as is presented in an examination of the records of surgery on the subject of compound fractures, gun-shot wounds, &c., and no kind of apology can be given for a persistence in that kind of surgery that has neither success, nor the comfort of patients to commend it. It is strange that surgeons tenaciously adhere to the doctrine of splints for fractures, when they admit that no apparatus of the kind is sufficient. But such is the hold which established custom makes, that even Mr. Maclise, after all the facts and strong declarations he has uttered, even after the demonstrations he has given of the utter inefficiency of all fracture apparatus, yet moves only in the periphery of the circle. He proposes further modifications of the splint machinery!

We think it is time that surgery should make a tangent from the well beaten circle to which we have referred.—The roller, properly understood, and properly applied, is the only agent that has yet been discovered, that will fulfil every indication in the treatment of fractures. It is the only one that is suitable for every form of fracture, from the simplest to those terrible injuries that involve joints, and is the only one that is adapted to all ages and conditions. Under its genial influence, the muscular sys tem is made to assume and retain its natural position and actions, and when this is accomplished, the broken bones are incapable of those displacements, which Mr. Maclise acknowledges are unavoidable with every species of splint apparatus. When the surgeon reduces a fracture, and gets the muscles of the limb under the control of the roller, he rests as secure as to the co-aptation of the fragments, as he does when the callus is fully formed. He knows, too, that no matter how badly comminuted the bones may be, nor how severe the contusions are, if there is any hope of a reparative process, the roller lights that hope with the strongest guarantee of fruition. There can be no suppuration in the medullary tissue of the bone, no suppurative action in the soft parts, no abscesses burrowing in every direction, no sloughs, no caries, nor any one of the other evils, which all surgeons admit are attendant upon every other form of treatment. But these are gains from the mere avoidance of evils—the positive advantages under the roller treatment are still greater.—The bandage prevents that lateral space which muscles require for contraction; and under its use patients may be taking exercise at an early stage of the treatment, while, under the use of the long splint, or double inclined plane, at the same period in the case, they would be confined to bed. The freedom from pain, too, is a matter of no small moment.

The duty of the surgeon is imperative—he is bound to make himself skilful in the use of the roller, in order to give those who rely on his judgment the best treatment the resources of his art can bring to bear upon the case. The skill can only be acquired by practice; the judgment must be formed upon the profoundest study of the subject, and upon critical observation. With such preparation as this, he will find that he has an agent of more inestimable value in the treatment of all fractures of the extremities, and gun-shot wounds, than all other things, for that object, that have ever been named. But it must ever be borne in mind, that an agent of such great powers for good, has equal capacity for evil, if improperly used. There is a world of difference between the administration of twenty grains of corrosive sublimate and the same amount of calomel. We can derive no benefit from anything whatever, except on the condition that it be used properly. The greatest errors prevail on the application of the roller; it is sometimes put on as a ligature, against which we have already quoted the admonitory voice of John Bell; it is often used without regard to the age, sex, and temperament of patients; and it is often put on so as to press unequally on different parts of the limb. To impress these truths upon the mind, we quote from the Dublin Hospital Gazette. Dr. Ferral reports a case of gangrene, and loss of a thumb, in a child, "from the application of a bandage, required in the adjustment of a fracture of the forearm." "A like result," says the London Lancet, "extending even to the loss of an entire limb, has frequently had its origin in a similar cause." We think our readers, when they examine the case, will say with us, that Dr. Ferral's was one of *misapplication* of the bandage, rather than the application of it. Dr. Ferral says: "The thumb suffered more severely, and was lost altogether. This is easily explained by the mode, so often adopted, of applying a bandage on the hand and forearm. A slit is made in the bandage, and through this slit the thumb is passed, in order to prevent the disturbance of the roller. In the present case, the thumb, as well as the whole hand and arm swelled after the application, and the thumb was consequently strangulated." No other result than this should have been looked for. Infants bear the roller as well as older persons, but it must be adapted to their age and condition. In all cases, for all ages, in applying the roller to the hand or arm, it is essential that the thumb and each finger be firmly bound with a narrow roller ; the roller may then be placed on the hand, and if it is properly done, no harmful result can follow.

The details of Dr. Caldwell's case on pages 116, 117, and 118, show the character of the duties involved in the application of the roller, in compound fracture of the thigh. In addition to those details, we quote from the first volume, quarto edition, of John Bell's surgery, a lucid account of the application of the roller. In the fourth discourse, we have a masterly treatise upon "the various uses of the bandage," constituting a monograph on the subject that is not surpassed by anything we know of in the literature of surgery. The surgeon who makes himself familiar with the "Discourse" referred to, will be thoroughly furnished for the practice of his profession in this department—without the knowledge contained in it, his surgery is crippled and crippling, no matter what his other resources may be. If we had to sum up, in a sentence, the duties of a student of surgery we should say to him : Study John Bell's "Discourse on the various uses of the bandage ;" learn to practice its principles, after you make yourself familiar with its use, you may then regard yourself qualified to practice in an extended field of surgery.

In this treatise, John Bell gives the following directions for the application of the roller: "It is obvious, that of all bandages the simple roller is best fitted for a diseased limb; but the moment you begin to apply this simple bandage, you will meet with unexpected difficulties; you will feel the necessity of use and practice towards rolling a limb with neatness and perfect *effect;* you will find yourselves awkward at first, and would almost believe, that a simple roller could never be made a perfect support to a diseased joint; you will perceive your bandages to be irregular from the first, and they will be slackened in a few hours. Practice will convince you, that the firmness and neatness of a bandage depends altogether upon these two points, first upon the TURNS succeeding each other in a regular proportion; and, secondly, upon making REVERSES, wherever you find any slackness likely to arise, from the varying form of the limb. Thus, in rolling from the foot to the ankle, leg, and knee, you must take care, first, that the turns, or, as the French call them, doloires, of the roller, lie over one another by just one-third of the breadth of the bandage; and, secondly, that at every difficult part, as over a joint, you turn the roller in your hand, make an angle, and lay the roller upon the limb with the opposite flat side toward it; you must turn the bandage so as to reverse it, making what the French call a Reversée of the roller, at the ankle, at the calf of the leg, and at the knee; wherever, upon making a turn of the roller, you perceive that it will fall slack, you make a *reverse* of the bandage, and at each reverse you put in a pin to prevent it falling down; you must be careful to roll your bandage from below upwards, and support the whole limb by a general pressure, that you may be able to support the diseasd part with a particular pressure," &c.

No man ever had a clearer conception of the powers and general use of the roller, than John Bell, and it is strange, that with all the knowledge he had of its use aad action, he did not more powerfully enforce its application to fractured limbs, because it is evident from what he says, that he knew the value of the roller in the treatment of fractures. He also knew its power over gangrene, for which Baron Larrey recommended his unguents.

It is interesting, however, to look at the compass of Bell's use of the bandage in surgery, and we take pleasure in giving a synopsis of his able and valuable views. He had a clear conception of the subject, and urged upon his pupils that, "a bandage is not merely useful in tying up a wound, but in accomplishing *many important operations in surgery, more interesting, indeed, than those which are done with the knife.* In wounds, in abscesses, in fistulas, in any general disease of a limb, bandaging is the chief operation of surgery; what the knife cures, it partly destroys; what the bandage cures, it saves." The latter remark should be written in letters of gold.

We have already quoted, from the first portion of John Bell's treatise, his commendation of the bandage in gunshot wounds. Its use in hemorrhage is urged with much judgment, as the reader will see from our quotation in the first portion of this paper. In abscesses, aneurisms, fistulas, "in a limb undermined with suppurations," John Bell urged the bandage with great eloquence. In reference to the latter, he says: "It is in a complicated case of a swelled and diseased limb that we are sensible of all the uses of a bandage, *which is a universal cure for all its disorders.* By the bandage we dissipate the leucophlegmatic swelling, abate the inflammation, prevent the extension of matter, lessen suppurating cavities, close the walls of fistulas, and procure the reunion of surfaces which have suppurated." In the same truthful and philosophic spirit, John Bell urges the bandage in varices, and in ulcers. In the latter, he says: "Let the limb be rolled from the very extremities of the toes, and then roll it as tight as you will, you can do no harm. * * * The bandage so rolled, by a skillful hand, is the only certain cure; neither ointments, nor mercurial preparations, nor sponges, nor leaden plates, will cure an ulcer—it is to be cured only by firm, equable, and perfect compression. * * * * There is no inflammation of the lower extremities, in which I do not experience the good effects of firm rolling." John Bell used the bandage for sprains, and dropsical effusions of joints, and knew its power over the absorbent system. He says, too: "By compression merely, ganglions may be obliterated, tumors of various kinds dissipated, and proud flesh or fungus suppressed," and he declared that many other things could be done by compression, which it was not his design to mention in his discourse.

We here close our remarks on the common surgery of fractures, and the treatment of those injuries by the roller. If what we have said, shall induce practitioners to pause before they remove a limb that may be saved; and in full confidence of the reparative powers of nature, under the supporting presence of the roller, they prefer an effort at saving, rather than a display of a set of amputating instruments; we shall be abundantly remunerated for the labor bestowed on this essay. And we shall greatly rejoice, if we have presented the subject in such a point of view as to induce the abandonment of all apparatus for fractures, except the simple roller, properly understood and applied. The physician who goes forth among his fellow men, equipped for the use of the roller with judgment and skill, may rest assured that he is prepared to be a benefactor to his species. We would not exchange what we have seen accomplished with the roller, for the knowledge that we were universally esteemed the first of amputators. Almost any one may cut off limbs; true science and skill alone can do the better part —save them.

In noticing the remark of Mr. Vincent, on the treatment of varicose ulcers by the roller, Mr. Critchett says: "If I had no other motive for publishing these lectures, a sufficient reason might, I think, be found in the manifest duty of endeavoring to counteract the tendency of such a remark from so high an authority, embalmed, as it is, among so many valuable and philosophic truths, and therefore the more calculated to exert a baneful influence upon the profession at large." If the remark of one able writer, on a single point in the treatment of one form of ulcer, could thus plead in behalf of Mr. Critchett's lectures on ulcers, surely the universal disasters that attend the ordinary treatment of fractures, may plead as a reason for calling attention to a method of treatment that is universally applicable, and which is unattended by any one of those evils that wait, as a train, upon the other management.

In a future number of this Journal, we shall continue our examination of some of the other uses of the bandage, beside those for fractures. During the composition of this paper, we have had several interesting cases that will serve to illustrate the beneficial effects of the roller, but we must reserve additional remarks for another number.

